# Fermentation of Murta (*Ugni molinae*) Juice: Effect on Antioxidant Activity and Control of Enzymes Associated with Glucose Assimilation

**DOI:** 10.3390/ijms242015197

**Published:** 2023-10-15

**Authors:** Natalia Escobar-Beiza, José R. Pérez-Correa, Wendy Franco

**Affiliations:** 1 Department of Chemical Engineering and Bioprocess, Pontificia Universidad Católica de Chile, Santiago 92101, Chile; nsescobar@uc.cl; 2 Departamento de Ciencias de la Salud, Carrera de Nutrición y Dietética, Pontificia Universidad Católica de Chile, Santiago 92101, Chile

**Keywords:** murta, lactic acid fermentation, antioxidant, polyphenols, enzymatic inhibition, α-amylase, α-glucosidase

## Abstract

Berries are rich in bioactive compounds, including antioxidants and especially polyphenols, known inhibitors of starch metabolism enzymes. Lactic acid fermentation of fruits has received considerable attention due to its ability to enhance bioactivity. This study investigated the effect of fermentation with *L. mesenteroides* of juice from the Chilean berry murta on antioxidant activity, release of polyphenols, and inhibitory activity against α-amylase and α-glucosidase enzymes. Three types of juices (natural fruit, freeze-dried, and commercial) were fermented. Total polyphenol content (Folin–Ciocalteu), antioxidant activity (DPPH and ORAC), and the ability to inhibit α-amylase and α-glucosidase enzymes were determined. Fermented murta juices exhibited increased antioxidant activity, as evidenced by higher levels of polyphenols released during fermentation. Inhibition of α-glucosidase was observed in the three fermented juices, although no inhibition of α-amylase was observed; the juice from freeze-dried murta stood out. These findings highlight the potential health benefits of fermented murta juice, particularly its antioxidant properties and the ability to modulate sugar assimilation by inhibiting α-glucosidase.

## 1. Introduction

Recently, the interest in functional foods and their potential health benefits has significantly grown. Among these, fermented fruit juices have emerged as a promising source of bioactive compounds with antioxidant and antidiabetic properties [1]. Fermentation, a natural process involving the conversion of sugars into other compounds by microorganisms, has been used for centuries to enhance the nutritional value, flavor, and preservation of various food and beverage products [2].

In lactic acid fermentation, lactic acid bacteria (LAB) convert sugars into organic acids and other molecules of interest. These fermentations not only yield unique flavors and textures but also enhance the bioactive profile of the fermented foods. Traditionally, lactic acid fermentation is related to milk-derived products, but recently, there has been an increasing interest in exploring other food matrices for fermentation, including fruit juices [3]. Antioxidants, crucial in protecting the body against oxidative stress and associated with chronic diseases, are key bioactive compounds in fermented fruit juices [4]. These molecules are of special interest since they neutralize harmful free radicals, reduce inflammation, and support overall health.

Additionally, the antidiabetic potential of fermented fruit juices has gained considerable attention [5,6]. Diabetes, a metabolic disorder characterized by elevated blood sugar levels, is a major global health concern. The natural compounds in fermented fruit juices, including polyphenols, dietary fiber, and organic acids, have been reported to exhibit antidiabetic effects by improving insulin sensitivity, regulating blood glucose levels, reducing the risk of diabetic complications, and inhibiting enzymes associated with sugar assimilation [7]. Managing postprandial hyperglycemia, a key factor in developing and progressing type 2 diabetes, remains challenging. Hence, there has been growing interest in exploring natural sources of bioactive compounds with potential antidiabetic properties. Fermented juices, rich in diverse phytochemicals and microbial metabolites, have emerged as promising candidates [8].

Various fruits, such as berries, grapes, pomegranates, and citrus fruits, have been used for fermentation, forming unique phytochemical compositions and enhancing bioactivity [9,10]. Moreover, using specific fermentation techniques and selecting appropriate microbial strains can further optimize the production of bioactive compounds and their health-promoting effects [11].

Argentina and Chile are rich in wild berries, such as murta (*Ugni molinae* Turcz), known for its high antioxidant capacity due to the presence of various bioactive compounds such as flavonoids and anthocyanins [11,12]. These compounds exhibit strong free radical scavenging activity, protecting cells and tissues from oxidative damage. On the other hand, other native berries have shown antidiabetic effects [13], raising the question of whether murta has similar properties. 

Therefore, this study aimed to investigate and compare the antioxidant properties of three different murta (*Ugni molinae* Turcz.) juices and evaluate their ability to control enzymes associated with glucose assimilation. This study assessed the variations in antioxidant activity among the different murta juice samples by measuring their scavenging capacity against free radicals and evaluating their total phenolic contents. Furthermore, the inhibitory effects of the juices on key enzymes involved in glucose assimilation, such as α-glucosidase and α-amylase, were investigated. 

## 2. Results and Discussion

### 2.1. Fermented Fruit Juices Characteristics

Three different murta juices (natural, commercial, and freeze-dried) were fermented with *Leuconostoc mesenteroides* (Accesion number OR395120), a BAL isolated from murta fruit flesh. Fermentation was carried out with and without adding glucose as a complementary carbohydrate source to aid the fermentation. Table 1 shows the principal characteristics of the fermented juices.

### 2.2. Total Phenolic Content (TPC) and Antioxidant Activity

Murta is a berry characterized by its high polyphenol content involved in various antioxidant mechanisms [14,15]. Hence, both concentrations of total polyphenols (TPC) and antioxidant activities were studied.

The TPC was determined with the Folin–Ciocalteu method [16]. Antioxidant activity was measured by the DPPH radical reduction capacity assay [17] and the ORAC [18] methods, where the IC50 concentration was determined. Table 2 shows the data related to these analyses.

#### 2.2.1. Total Polyphenol Content (TPC) 

Compared with the control juices, all fermented murta juices showed a significant increase (*p* < 0.05) in the total polyphenolic content, except for a decrease in the commercial fermented fruit juice (JCF) of approximately 71 mg GAE/mL. After fermentation with *L. mesenteroides* OR395120, it was found that JNF + G, JNF, and JCF + G were the juices with the highest TPC values, with 635 ± 5, 618 ± 1, and 593 ± 2 mg GAE/L, respectively.

In general, the release of polyphenols by LAB is limited by the composition of the fermenting food [19], which can be observed in the difference in concentration of the molecules depending on the juice formulations. JLF showed higher TPC than nonfermented juice (JLF + G: 84.2%, JLF: 145%). In the fruit matrix, soluble dietary fibers interact with polyphenols, reducing their bioavailability and bioactivity [20]. LAB fermentations promote the release of polyphenolic compounds by weakening the interactions between polyphenols and other food components [21], which impacts the overall TPC [22], as evidenced in this research by the significant increases in the TPC after the fermentation of murta juices. Polyphenol release after fermentations with *L. mesenteroides* has been reported for various juices such as purslane (*Portulaca oleracea* L.) with an increase in the TPC of 270 mg GAE/L after a 36-h fermentation with the strain *L. mesenteroides* OP9 [23]. Increases of 0.5 mg GAE/mL and 0.4 mg GAE/mL in fermented cabbage and tomato juices, respectively, were observed in fermentations with the strain MKSR [24]. 

The decrease in the TPC in JCF may be due to the depolymerization of macromolecular polyphenols and the conversion of these into other molecules that can decrease the TPC values [25]. Some LABs can consume phenolic compounds in their metabolism [26]. Moreover, the decrease might be associated with the higher lactic acid content. The total phenolic content in a substance can be reduced in the presence of lactic acid due to several factors related to the chemical interactions between these compounds. As an organic acid, lactic acid can promote hydrolysis and degradation of phenolic compounds [27]. JCF showed the highest lactic acid concentrations, while lower concentrations in the other fermented juices were observed, suggesting a correspondence between the quantity of lactic acid and the degradation of polyphenols. 

Glucose addition affected in different forms the TPC of the fermented juices. For JCF + G, a significant increase (*p* < 0.05) in the total polyphenol concentration compared with the unfermented juice was observed. Although increases were observed when glucose was added to the natural juice, this was not significant. 

#### 2.2.2. DPPH Free Radical Scavenging Method 

Regarding the DPPH assay, the characterization of the juices before fermentation showed that the nonfermented juice with the lowest IC50 value was JC with 160 ± 5 mg GAE/L, followed by JN with 272 ± 10 mg GAE/L and then JL, which only exhibited 32.6% inhibition of radical activity. According to the literature, murta has a remarkable capacity for capturing DPPH free radicals. The best results have been obtained through extractions with solvents such as ethanol, methanol, and water [28,29,30], and for murta juice, a DPPH value of 328 ± 50 μg TE/mL [31] has been reported. The commercial and natural murta juices in this research had lower IC50 values, reflecting the raw materials’ good initial antioxidant capacity.

After fermentation, significantly lower (*p* < 0.05) IC50 values were obtained in all murta juices. This means the fermented samples require a lower concentration of antioxidant agents participating in proton exchange reactions (compared with their initial state) to inhibit 50% of the radical activity. JCF juices obtained the lowest IC50 values with 74 ± 10 and JCF + G with 89 ± 4 mg GAE/L, while the control juices had the highest. Considering the total content of polyphenols present in the juices, it can be observed that most of the juices meet the minimum amount required to inhibit more than 50% of the activity of DPPH free radicals, which reflects their high antioxidant capacity and the positive impact of lactic acid fermentation.

In general, the *L. mesenteroides* OR395120 isolate positively enhanced the antioxidant activity of fermented foods, and our results are comparable with those reported for commonly used LAB. For example, the IC50 reduction in JCF juice is similar to that yielded by fermentation with *Lactobacillus plantarum* 90 in apple juice after a 48-h fermentation [4]. Moreover, the percentage of inhibition of the DPPH radicals of JCF juice is similar to that of ethanolic extracts of murta leaves, with a reported value of 92.6% [32]. The JNF juice exhibited similar behavior to the fermentations of strawberry juice for 48 h with *L. plantarum* and *L. acidophilus* [33]. Finally, the freeze-dried fermented juices had low inhibitory capacities. Still, they showed the greatest increase in DPPH free radical scavenging, a similar behavior reported for 36-h fermentations of mulberry juice with strains of *L. plantarum*, *L. acidophilus*, and *L. paracasei* [34].

#### 2.2.3. Oxygen Radical Absorbance Capacity (ORAC) 

Before fermentation, the commercial juice, JC, had the highest ORAC (65 ± 2 μmol TE/mL), followed by JL (62 ± 3 μmol TE/mL), and finally JN (44 ± 2 μmol TE/mL). It has been previously reported for ripe murta berry an ORAC of 107.7 μmol TE/g wet weight [11,35,36] and 3300 ± 200 μmol Trolox/g for an ethanolic extract of the berry [37]. In comparison, 1 mL of any of the murta juices considered in this research has slightly lower values than one gram of wet murta berries. However, this can be due to the dilution of polyphenolic compounds caused by the juice formulations.

The ORAC significantly increased (*p* < 0.05) in all juices after fermentation. This increase has also been reported in fermented vegetables with *L. mesenteroides*, such as sauerkraut [38]. *L. mesenteroides* increases the bioavailability of polyphenolic compounds [39] through hydrolysis, producing low-molecular-weight phenolic acids [40]. Additionally, *L. mesenteroides* produces various metabolites, such as peptides, that augment the antioxidant activity of fermented foods [41]. Glucose favored the release of polyphenols with a high capacity to absorb oxygen radicals since the fermented juices supplemented with glucose (+G) showed the highest ORACs. Among them, JCF + G showed the highest ORAC (261 ± 30 μmol TE/mL), followed by JLF + G (253 ± 30), and finally JNF + G (201 ± 5 μmol TE/mL). These values are similar to calafate (256.6 μmol TE/g), a native berry widely known for its high antioxidant activity [11]. Although fermented murta juices without glucose supplementation exhibitedlower values (*p* < 0.05), their ORACs (93.2–160.7 μmol TE/mL) are high compared to other nonfermented fruit juices that stand out for their antioxidant capacities, such as pomegranate (8.8–11.8 mmol TE/L [42]) and purple grape (4.5–11.5 μmol TE/mL [43]).

Due to the LAB enzymatic activity, several polyphenols are released from the plant material during fermentation [44]. LAB has a wide array of enzymes that can convert, for example, hydroxycinnamic and hydroxybenzoic acids into smaller low molecular bioactive phenolic compounds [44,45].

The release of these molecules has been associated with increased antioxidant activity when berries are fermented. Additionally, a correlation between the presence of certain molecules and bioactivity has been reported. For example, in mulberry juice fermentations by LAB, a strong positive correlation was observed between antioxidant activity (DPPH, ABTS, and FRAP) and the levels of cyanidin 3-O-glucoside, cyanidin 3-O-rutinoside, total phenolic content, and total anthocyanin content [22]. The authors suggested that the observed increase in antioxidant activity may be related to the ability of LAB to metabolize polyphenols during fermentation. In another study, Suthanthangjai et al. (2013) [46] reported that the use of LAB to ferment blueberry juice resulted in the metabolization of malvidin-3-glucosides into syringic acid, protocatechuic acid, gallic acid, and other phenolic acids and, at the same time, improved the juice antioxidant activity [47]. Furthermore, Wu et al. (2021) [48] fermented blueberry and blackberry juice using four different LABs and found that the content of cyanidin-3-glucoside and peonidin-3-glucoside significantly decreased during the fermentation process. At the same time, the antioxidant activity of the juice increased.

Murta is rich in anthocyanins and flavonoids (Table 3), and therefore, it is expected that the fermentation of the murta juices with *L. mesenteroides* OR395120 might also influence the behavior of the polyphenols, impacting the antioxidant activity. Lactic acid bacteria can demethylate the glycosidic bonds of anthocyanins to produce low-molecular-weight polyphenols [49]. Avila et al. (2009) [50] found that ferulic acid can be produced upon the cleavage of B/C rings in cyanidin 3-O-glucoside, while phenolic acids (e.g., gallic and syringic) can be produced by the degradation of malvidin anthocyanins.

### 2.3. Enzyme Inhibition Activities

α-Glucosidase and α-amylase enzymes play an important role in sugar assimilation. Therefore, its inactivation or inhibition is an important alternative to control, for example, type 2 diabetes. Acarbose is commonly used to treat the problem. Despite its effectiveness, it shows adverse effects such as toxic hepatitis and unwanted gastrointestinal ailments [51]. Consequently, various research studies have been conducted to find natural supplements with similar effects. Berry juices have been reported as potential inhibitors of α-amylase and α-glucosidase, representing a functional food option to acarbose [52,53]. The antidiabetic properties of fruit juices (natural and fermented) have been associated with their antioxidant activity [5] and polyphenol composition [22]. Since fermented murta juices showed higher antioxidant and polyphenolic content than unfermented juices, their inhibitory activity against these enzymes was assessed.

#### 2.3.1. Inhibitory Activity of α-Amylase Enzyme 

α-Amylase is an endoglycosidase enzyme involved in carbohydrate metabolism, such as starch, catalyzing the hydrolysis of α (1 → 4) glucosidic bonds and converting them into oligosaccharides [54]. In the small intestine, these are converted into monosaccharides, which are absorbed and transported through the blood, increasing glucose levels. Consequently, controlling carbohydrate digestion is crucial to avoid or delay the onset of type 2 diabetes mellitus [55].

**Figure 1:**
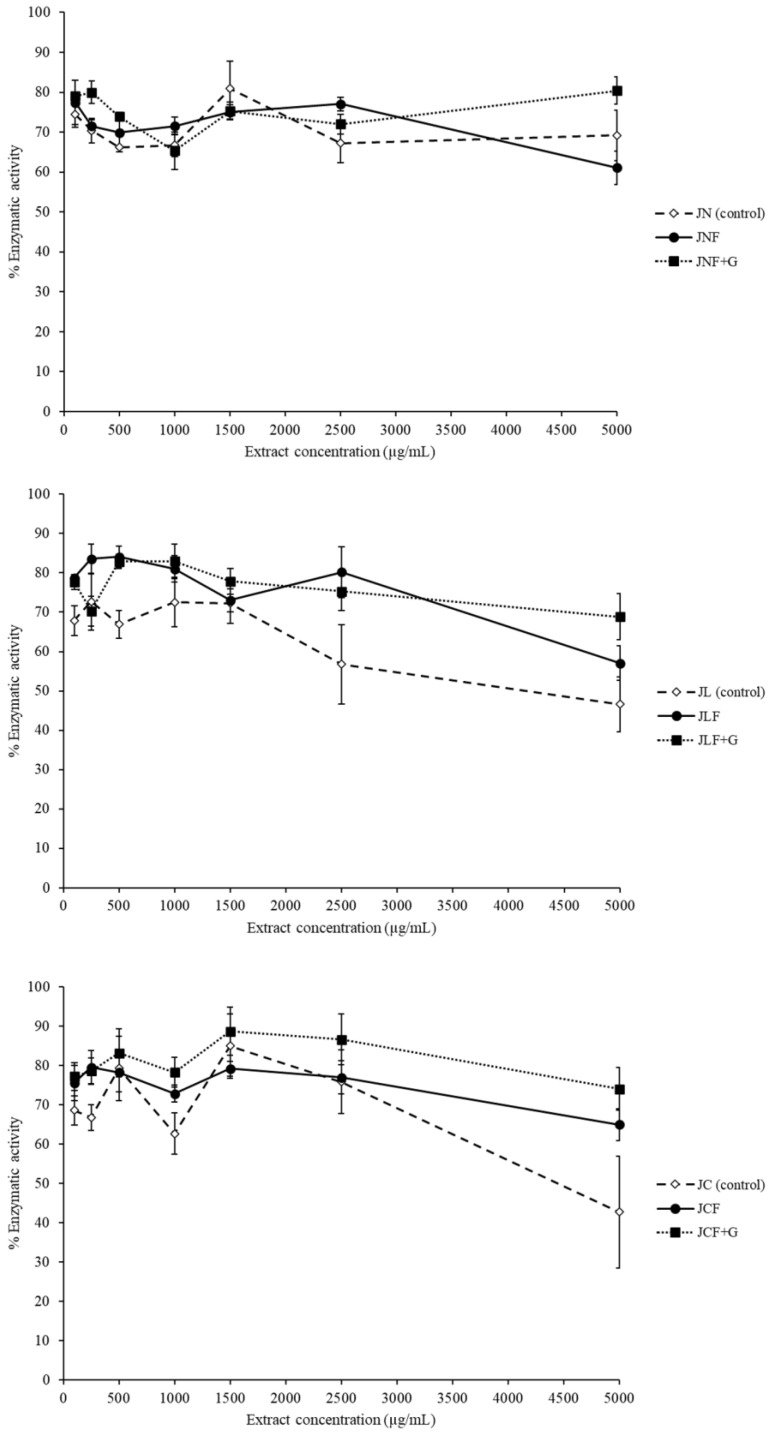
Inhibition of α-amylase by murta juices. JN: nonfermented natural juice; JNF: fermented natural juice; JNF + G: fermented natural juice with added glucose; JL: nonfermented freeze-dried juice; JLF: fermented freeze-dried juice; JLF + G: fermented freeze-dried juice with added glucose; JC: nonfermented commercial juice; JCF: fermented commercial juice; JCF + G: fermented commercial juice with added glucose.

There was no significant inhibitory capacity against α-amylase in the fermented murta juices (Figure 1), neither in the juices with or without glucose addition. Moreover, only the unfermented juices JL and JC showed a measurable IC50 of 4159 ± 10 and 4450 ± 20 μg/mL, respectively. These values are much higher than the IC50 of 32 ± 2 μg/mL we obtained for acarbose. Previous studies have reported that ethanolic extracts of fruits and leaves of murta possess excellent inhibitory capacity against α-amylase (IC50 between 80 and 100 μg extract/L) [29]. Hence, juice preparation and fermentation seemed to induce the degradation or deactivation of α-amylase inhibitory compounds. The negative impact of *L. mesenteroides* OR395120on α-amylase inhibition has been previously reported in kimchi [56].

#### 2.3.2. Inhibitory Activity of α-Glucosidase 

The α-glucosidase enzyme plays an important role in blood glucose control and postprandial hyperglycemia, which occurs after food intake. This is because it hydrolyzes oligosaccharides in the small intestine [57]. This enzymatic activity can be reduced by consuming inhibitory compounds with a high affinity for the enzyme’s active site and directly competing with carbohydrates [58], thus reducing the likelihood of developing type 2 diabetes.

The literature has reported the significant inhibitory capacity of α-glucosidase by ethanolic extracts of murta fruit, which have an IC50 value of 69 ± 5 mg extract/L [29]. However, to date, the inhibitory capacity of this enzyme has not been studied in other formats, such as fruit, pulp, or juice.

As shown in Table 4, *L. mesenteroides* OR395120 significantly improved (*p* < 0.05) the IC50 values of most fermented juices.

**Figure 2:**
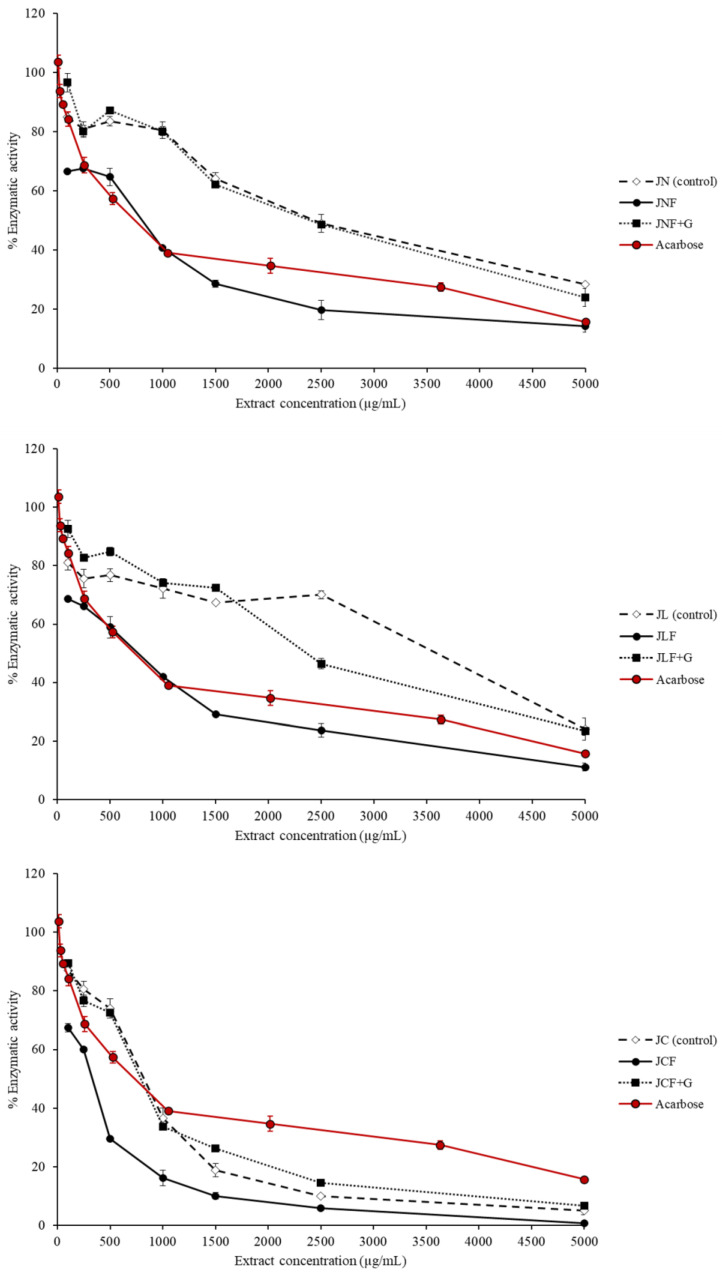
Inhibition of α-glucosidase by murta juices. JN: nonfermented natural juice; JNF: fermented natural juice; JNF + G: fermented natural juice with added glucose; JL: nonfermented freeze-dried juice; JLF: fermented freeze-dried juice; JLF + G: fermented freeze-dried juice with added glucose; JC: nonfermented commercial juice; JCF: fermented commercial juice; JCF + G: fermented commercial juice with added glucose.

The JCF juice exhibited an IC50 of 332 ± 4 μg/mL, which is 50% smaller than the acarbose control, while JNF and JCF + G juices showed IC50 values a bit higher than acarbose (699 ± 4 μg/mL). Nevertheless, it can be observed in Figure 2 that after 1000 μg/mL, all commercial juices show higher inhibitory capacity than acarbose (in red). Glucose supplementation is detrimental to the α-glucosidase inhibition capacity of fermented juices since it may increase the liberation of soluble dietary fibers or other compounds during fermentation, affecting the interaction between polyphenols and α-glucosidase [59].

Other strains of *L. mesenteroides* that increase the α-glucosidase inhibitory capacity of the unfermented matrix have been isolated from kimchi [56] and Nile tilapia fish [60]. Polyphenols and certain exopolysaccharides liberated during fermentation have been associated with these increments [41,56,61]. Liu et al. (2020) [62] reported that polyphenols present in *Loniccera caerule* berries, such as cyanidin-3-glucoside, catechins, and chlorogenic acid, can inhibit pancreatic α-amylase by changes in the protein conformation and the formation of polyphenol–enzyme interactions. In another study, polyphenolic extracts of the same berry showed antidiabetic properties, including inhibition of α-amylase (IC50 ranges from 2380 to 5080 μg/mL) and α-glucosidase (IC50 ranges from 1130 to 2120 μg/mL). These extracts, similar to murta, are composed of anthocyanins belonging to the cyanidin, petunidin, and peonidin families [63], which suggests that these molecules might be associated with the enzymatic inhibition pathways. Li et al. (2023) [22] reported IC50 ranging from 16 to 25 μg/mL for different mulberry juices. In our study, IC50 values for the fermented juices were lower than those reported in the literature, with values of 805 μg/mL for the natural fermented juice, 761 μg/mL for the freeze-dried fermented juice, and 332 μg/mL for the commercial fermented juice. The above suggests that the changes in TPC and probably the phenolic profile resulting from the fermentation process have an important impact on the dosage needed to control the enzyme.

## 3. Materials and Methods

### 3.1. Chemicals and Reagents

Ascorbic acid (L(+)-ascorbic acid powder) was purchased from AppliChem (Darmstadt, Germany). Glucose (D-glucose) was obtained from Winkler (Santiago, Chile). The De Man, Rogosa, and Sharpe (MRS) agar was purchased from OXOID (Basingstoke, Hampshire, England). The Folin–Ciocalteu reagent, sodium carbonate (Na_2_CO_3_), monopotassium phosphate (KH_2_PO_4_), dipotassium phosphate (K_2_HPO_4_), methanol, sodium chloride (NaCl), starch, and sodium and potassium tartrate tetrahydrate (C_4_H_4_KNaO_6_·4H_2_O) were obtained from Merck (Darmstadt, Germany). Gallic acid (3,4,5-trihydroxybenzoic acid) was purchased from Acros Organics by Thermo Scientific (Waltham, MA, USA). The DPPH reagent (2,2-diphenyl-1-picrylhydrazil), fluorescein sodium salt, 2,2′-azo-bis(2-amidino-propane) dihydrochloride (AAPH), Trolox, α-amylase from porcine pancreas enzyme, 3,5-dinitrosalicylic acid (DNS), sodium hydroxide (NaOH), acarbose, α-glucosidase enzyme, and 4-nitrophenyl α-D-glucopyranoside (PNPG) were obtained from Sigma-Aldrich (Saint Louis, MO, USA). 

### 3.2. Juices Preparation

#### 3.2.1. Natural Juice

Murta fruits were processed in a blender (Oster) until pulp was obtained. Then, 500 mL of commercial mineral water was added to 125 g of the pulp, equivalent to a ratio of 1:4, respectively [64]. The ratio was selected to standardize total soluble solids in the samples and prepare a light beverage comparable with the commercial murta juice. Large particles were removed from the diluted pulp using sterilized gauze. The juice (diluted pulp [64]) was then pasteurized in a water bath (WNB 14, Memmert, Schwabach Germany) at 75 °C for 30 min and cooled at room temperature.

#### 3.2.2. Commercial Juice

Commercial murta juice (Machitún, Cañete, Bío Bío) was purchased in a local store. The juices were kept closed and at room temperature until experimentation. The expiration date of the juices showed a remaining shelf-life of about one year. 

#### 3.2.3. Freeze-Dried Juice

Commercial freeze-dried murta powder from Nahuelbuta (Temuco, La Araucanía Region, Chile) was obtained and frozen at −40 °C until use. For juice preparation, 7 g of the powder was dissolved in 500 mL of mineral water (1.4% *w*/*v*) [65,66]. The solid sediments were removed from the juice using sterilized gauze. The juice was then pasteurized in a water bath (WNB 14, Memmert, Schwabach, Germany) at 75 °C for 30 min and allowed to cool at room temperature.

To avoid oxidative damage, all juices were supplemented with 0.05% *w*/*v* ascorbic acid [67] before pasteurization. After pasteurization, the juices were standardized to a pH of 5.0 and separated into two batches. The first was directly used, while the second was supplemented with 2% (*w*/*v*) glucose to boost lactic acid fermentation. Table 5 shows the chemical characteristics of the juices. 

### 3.3. Juices Fermentation

Prepared juices were fermented with *L. mesenteroides* OR395120, previously isolated from murta pulp. Fresh active cultures were added (2% *v*/*v*) to each juice at a 6.2 log CFU/mL concentration and mixed. The mixtures were incubated at 30 ± 2 °C and 100 rpm (SI500 orbital incubator) for two days. Then, the juices were analyzed to determine the glucose and lactic acid concentration using a photometer Analyzer Y-15 (Biosystem, Barcelona, Spain). The final LAB population was determined by plating in MRS agar.

### 3.4. Total Polyphenols Content (TPC) 

The content of total phenolic compounds was estimated using the Folin–Ciocalteu method [16]. Before the analysis, the samples were filtered with a 0.22 µm filter, obtaining a remnant sample free of microorganisms. 

First, the diluted reagent was prepared at a 1:1 volumetric ratio of the Folin–Ciocalteu reagent with distilled water. Briefly, 0.5 mL of juice samples was placed in test tubes, and 3.75 mL of distilled water was added. Then, 0.25 mL of the diluted Folin–Ciocalteu reagent was added, and the sample was homogenized at medium speed with a vortex mixer. An amount of 0.5 mL of the 10% *w*/*v* sodium carbonate solution was added to the homogenized samples and left to rest for one hour at room temperature in dark conditions. Subsequently, the absorbance of the samples was measured at a wavelength of 765 nm in a Genesys 150 UV–Vis spectrophotometer (Thermo Fisher Scientific, Waltham, MA, USA). A calibration curve with known concentrations of gallic acid was used for TPC quantification. Pure distilled water was used as a blank sample. The results were expressed in units of mg of gallic acid equivalents (GAE) per liter of juice. 

### 3.5. Antioxidant Activity

The antioxidant activity of the juices was determined by 2,2-diphenyl-1-picrylhydrazyl DPPH and the oxygen radical absorbance capacity (ORAC) analysis. The samples were previously filtered with a 0.22 µm filter, as mentioned in Section 3.4.

#### 3.5.1. DPPH Radical Scavenging Activity

The methodology reported by Erpel et al. (2021) [17] was followed with some modifications. First, a 39.4 mg/L DPPH in a methanol solution was prepared and stored at 4 °C until use. Subsequently, five methanolic dilutions of the juice samples at different concentrations were prepared. Aliquots of 0.1 mL of each diluted sample were taken, and each was mixed with 3.9 mL of the DPPH solution. The samples were homogenized with a vortex shaker at medium speed and left to rest for 30 min at room temperature in dark conditions. Then, the absorbance of each sample was measured at 517 nm in a Genesys 150 UV–Vis spectrophotometer (Thermo Fisher Scientific, Waltham, MA, USA). The results were expressed in IC50 of mg gallic acid equivalents (GAE) per mL of juice, and the percentage of inhibition of DPPH radicals was calculated according to

$$\%Inhibition=\left(1-\frac{Abs_{blank}}{Abs_{sample}}\right)\cdot 100$$

A 3.9 mL pure methanol sample was used as a blank, and 3.9 mL of the methanolic DPPH solution was used as a negative control.

#### 3.5.2. Oxygen Radical Absorbance Capacity (ORAC)

The methodology reported by Huang et al. (2022) [18] with some modifications was followed. A 75 mM concentration PBS buffer solution was prepared using K_2_HPO_4_ and KH_2_PO_4_ salts, and the pH was adjusted to 7.4. This buffer was then used to prepare fluorescein solutions at 55 nM and AAPH at 153 mM.

In a dark 96-well microplate, 25 μL of the sample was added to 250 μL of the fluorescein solution and incubated for 30 min at 37 °C. Then, 25 μL of fresh AAPH solution was added, which started the oxidation reaction. Fluorescence was measured every minute for one hour at 37 °C in a Synergy HTX multimode microplate reader (BioTek Instruments, Winooski, VE, USA) at 485 nm excitation and 528 nm emission wavelengths. The blank of the analysis was the PBS buffer solution.

Gen 5 software (BioTek Instruments, Winooski, VE, USA) was used to record the data and determine the areas under the curve (AUCs) of the samples, which were calculated with the following equation:

$$Net\;AUC=AUC_{sample}-AUC_{blank}$$

The calibration curve was determined with solutions containing 1.3 and 10.5 mg/L of Trolox prepared in PBS buffer. The results were expressed in μmol of Trolox equivalents per mL of juice.

### 3.6. Inhibition of Carbohydrate Hydrolytic Enzymes

The inhibitory activity of the α-amylase and α-glucosidase enzymes of the fermented murta juices was determined following the methodology described by Erpel et al. (2021) [17] with some modifications.

#### 3.6.1. Pretreatment of Samples

Unfermented and fermented juices were frozen at −80 °C and freeze-dried in a BK-FD12PT model vacuum freeze-dryer (BIOBASE, Wolfenbüttel, Germany) for three days. The freeze-dried powders obtained were kept in a desiccator with cobalt chloride-free orange silica gel until a constant weight was obtained and stored at −40 °C in hermetic containers until use. In this investigation, on average, 0.8 mg of freeze-dried powder was obtained from 1 mL of murta juice. Subsequently, these solid samples were resuspended in the respective buffer solutions for each enzymatic analysis.

#### 3.6.2. Anti α-Amylase Activity

A buffer solution containing 20 mM phosphate and 6 mM NaCl was prepared, and the pH was adjusted to 6.9. A buffer solution with 0.5 mg/mL of α-amylase from porcine pancreas and 0.5% *w*/*v* starch was prepared. The freeze-dried samples were diluted between 100 and 5000 μg/mL. A DNS solution prepared following Erpel et al. (2021) [17] was used as a colorimetric reagent. For this solution, 5 g of the DNS reagent, 150 g of C_4_H_4_KNaO_6_·4H_2_O, and 8 g of NaOH were added to 400 mL of distilled water. The mixture was dissolved at 70 °C with a hot plate and a magnetic stirrer and allowed to cool to make up to 500 mL with distilled water.

Aliquots (100 μL) were taken for each sample dilution, and 100 μL of the 0.5% *w*/*v* starch solution was added. The mixture was incubated at 25 °C for 10 min in a WNB 14 thermoregulated bath. Then, 100 μL of the enzyme was added and incubated at 25 °C for 10 min. Subsequently, 200 μL of DNS was added and incubated in a thermoregulated bath at 100 °C for 5 min. Samples were allowed to cool at room temperature, and 50 μL aliquots were transferred to a 96-well microplate. These samples were diluted with 200 μL of distilled water.

Finally, the absorbance was measured at a wavelength of 540 nm in an Infinite M200 Pro microplate reader (TECAN, Männedorf, Switzerland). The samples without enzymes were used as blanks, while for the negative controls, the samples were replaced by phosphate buffer. Additionally, acarbose was used as a positive control. The enzymatic activity was calculated with the following equation:

$$\%Activity=\frac{Abs_{sample}-Abs_{blank}}{Abs_{control}}\cdot 100$$

Results were expressed as IC50 of μg of freeze-dried juice per mL. 

#### 3.6.3. Anti α-Glucosidase Activity

A 100 mM phosphate buffer solution adjusted to pH 6.9 was prepared. With this solution, 0.1 U/mL of α-glucosidase and 5 mM of PNPG solution were prepared. Moreover, dilutions of the freeze-dried powders of the samples were made at concentrations between 100 and 5000 μg/mL.

In a 96-well microplate, 50 μL of the sample and 50 μL of PNPG were added. Then, the microplate was incubated at 37 °C for 5 min in an Infinite M200 Pro microplate reader. Subsequently, 100 μL of α-glucosidase was added to start the enzymatic reaction. The microplate reader took absorbance readings at a wavelength of 405 nm every 3 min for 30 min at 37 °C. Like in the previous analysis, the samples without enzymes were used as blank, the phosphate buffer as a negative control, and acarbose as a positive control. The percentage of enzymatic activity and the IC50 factor for each dilution of the samples were also determined.

### 3.7. Statistical Analysis

All the experiments were carried out in triplicate in two experimental runs. The data were expressed as mean ± standard deviation. A one-way analysis of variance (ANOVA) was performed to determine significant differences. The post hoc *t*-test with Bonferroni correction was applied for multiple comparisons. Statistically significant differences were considered at *p* values < 0.05.

## 4. Conclusions

Fermentation of the three murta juices with *L. mesenteroides* OR395120 significantly improved the TPC of unfermented juices, especially those made with freeze-dried murta. This improvement was consistent with the increases in DPPH and ORAC analysis, reflecting a positive impact of fermentation on the antioxidant capacity of murta juices. The inhibitory activity against α-glucosidase was also improved by fermentation, with IC50 values comparable with those of acarbose. JCF showed the highest α-glucosidase inhibitory activity, although its TPC was reduced during fermentation. Fermentation negatively affects the capacity of the murta juices to inhibit α-amylase. In general, the bioactive properties of murta have been reported for untreated fruit or solvent extracts. To our knowledge, this is the first time TPC, antioxidant activity, and inhibition of enzymes have been reported for fermented murta products. This opens an opportunity to investigate the feasibility of developing natural bioactive products. 

## Figures and Tables

**Table 1:** Characteristics of fermented murta juices.

Juice Type	Glucose (g/L)	Lactic Acid (mg/L)	*L. mesenteroides* (Log CFU/mL)
JN	4.7 ± 0.2 ^a^	22.1 ± 0.1 ^c^	BDL
JNF	1.2 ± 0.1 ^c^	30.4 ± 0.2 ^b^	6.8 ± 0.1 ^a^
JNF + G	2.80 ± 0.04 ^b^	47.9 ± 0.1 ^a^	6.5 ± 0.2 ^b^
JL	0.77 ± 0.02 ^a^	ND	BDL
JLF	0.1 ± 0.1 ^c^	13.5 ± 0.2 ^b^	6.7 ± 0.2 ^a^
JLF + G	0.57 ± 0.04 ^b^	21.7 ± 0.1 ^a^	6.9 ± 0.2 ^a^
JC	3.0 ± 0.1 ^a^	22.3 ± 0.1 ^c^	BDL
JCF	1.74 ± 0.03 ^c^	31.8 ± 0.1 ^b^	6.7 ± 0.2 ^a^
JCF + G	2.1 ± 0.1 ^b^	20.6 ± 0.2 ^a^	6.8 ± 0.2 ^a^

Data are presented as the mean ± standard deviation of three replicates and two independent trials. A different lowercase letter in the superscript attached to the standard deviations represents significantly different values in the respective column. BDL: below detection limit; JN: not fermented natural juice; JNF: fermented natural juice; JNF + G: fermented natural juice with added glucose; JL: nonfermented freeze-dried juice; JLF: fermented freeze-dried juice; JLF + G: fermented freeze-dried juice with added glucose; JC: nonfermented commercial juice; JCF: fermented commercial juice; JCF + G: fermented commercial juice with added glucose.

**Table 2:** Total phenolic content and antioxidant activity of the studied murta juices.

Juice Type	TPC (mg GAE/L)	DPPH IC50 (mg GAE/L)	DPPH % Inhibition	ORAC (µmol TE/mL)
JN	467 ± 5 ^d^	272 ± 10 ^a^	71 ± 3 ^e^	44 ± 2 ^h^
JNF	618 ± 1 ^a^	123 ± 5 ^c,d^	80 ± 3 ^d^	93 ± 4 ^f^
JNF + G	635 ± 5 ^a^	136 ± 1 ^c^	74 ± 3 ^e^	201 ± 5 ^c^
JL	131 ± 2 ^g^	N.D.	33 ± 1 ^h^	62 ± 3 ^g^
JLF	322 ± 1 ^e^	115 ± 2 ^d^	59.2 ± 0.1 ^f^	161 ± 30 ^d^
JLF + G	242 ± 1 ^f^	126 ± 3 ^c,d^	53.2 ± 0.4 ^g^	253 ± 30 ^b^
JC	550 ± 3 ^c^	160 ± 5 ^b^	86.6 ± 0.4 ^c^	65 ± 2 ^g^
JCF	479 ± 3 ^d^	74 ± 10 ^e^	92.4 ± 0.2 ^a^	135 ± 30 ^e^
JCF + G	593 ± 2 ^b^	89 ± 4 ^e^	87 ± 1 ^b^	261 ± 30 ^a^

Data are represented as the mean ± standard deviation of three replicates and two independent runs. A different lowercase letter in the superscript attached to the standard deviations represents significantly different values in the respective column. N.D.: not detected; JN: nonfermented natural juice; JNF: fermented natural juice; JNF + G: fermented natural juice with added glucose; JL: nonfermented freeze-dried juice; JLF: fermented freeze-dried juice; JLF + G: fermented freeze-dried juice with added glucose; JC: nonfermented commercial juice; JCF: fermented commercial juice; JCF + G: fermented commercial juice with added glucose.

**Table 3:** Main bioactive compounds reported for murta berry.

Bioactive Compound	
Anthocyanins	Delphinidin Cyanidin Petunidin Peonidin Malvidin
Flavonoids	Quercertin Rutin Myricetin Kaempferol Luteolin Catechin
Hydroxycinnamic acids	Caffeic acid
Hydroxybenzoic acids	Gallic acid

(Adapted from Vega-Galvez, Rodriguez, and Stucken, 2021 [12]).

**Table 4:** Inhibitory activity of the α-glucosidase enzyme of the studied murta juices.

Juice Type	IC50 (µg/mL) α-Glucosidase
JN	2430 ± 10 ^c^
JNF	805 ± 20 ^d^
JNF + G	2390 ± 20 ^c^
JL	3588 ± 20 ^a^
JLF	760 ± 20 ^e^
JLF + G	2663 ± 20 ^b^
JC	820 ± 10 ^d^
JCF	332 ± 4 ^g^
JCF + G	790 ± 10 ^e^
Acarbose (control)	699 ± 4 ^f^

The data are presented as the mean ± standard deviation of three replicates and two independent trials. A different lowercase letter in the superscript attached to the standard deviations represents significantly different IC50 values. JN: nonfermented natural juice; JNF: fermented natural juice; JNF + G: fermented natural juice with added glucose; JL: nonfermented freeze-dried juice; JLF: fermented freeze-dried juice; JLF + G: fermented freeze-dried juice with added glucose; JC: nonfermented commercial juice; JCF: fermented commercial juice; JCF + G: fermented commercial juice with added glucose.

**Table 5:** Characterization of control murta juices.

Juice Type	pH	TSS	TTA (% Ascorbic Acid)	Glucose (g/L)	Fructose (g/L)
JN	3.5 ± 0.1 ^b^	1.7 ± 0.4 ^a^	0.09 ± 0.02 ^a^	4.7 ± 0.1 ^a^	2.8 ± 0.1 ^b^
JL	4.8 ± 0.1 ^a^	1.6 ± 0.1 ^a^	0.06 ± 0.02 ^a^	0.77 ± 0.02 ^c^	1.47 ± 0.04 ^c^
JC	3.6 ± 0.1 ^b^	1.7 ± 0.3 ^a^	0.09 ± 0.03 ^a^	3.0 ± 0.1 ^b^	3.8 ± 0.1 ^a^

The data are presented as the mean ± standard deviation of three replicates and two independent trials. A different lowercase letter in the superscript attached to the standard deviations represents significantly different values in the respective column. TSS: total suspended solids; TTA: total titratable acidity; JN: nonfermented natural juice; JC: nonfermented commercial juice; JL: nonfermented freeze-dried juice.

## Data Availability

The authors declare that the data supporting the findings of this study are available within the paper. Should any raw data files be needed in another format, they will be available from the corresponding author upon reasonable request.
